# Association Between Cancer Treatment History and Coronary Inflammation

**DOI:** 10.1016/j.jacadv.2025.102333

**Published:** 2025-11-19

**Authors:** Nariko Tsukamoto, Ayako Kunimura, Shimpei Kuno, Wataru Suzuki, Kazuhiro Naito, Hirohiko Ando, Yasushi Suzuki, Tetsuya Amano

**Affiliations:** Department of Cardiology, Aichi Medical University, Nagakute, Aichi, Japan

**Keywords:** cardiovascular disease, coronary computed tomography angiography, fat attenuation index, malignancy, peri-coronary adipose tissue

## Abstract

**Background:**

Previous studies have established a strong link between a cancer history and an increased risk of cardiovascular events.

**Objectives:**

This study aimed to determine whether a cancer history is independently associated with coronary inflammation, a key driver of atherosclerotic plaque development.

**Methods:**

This study included 1141 patients who underwent coronary computed tomography angiography from 2017 to 2018. We divided the patients into 2 groups based on the cancer history: 953 noncancer patients and 188 cancer patients. Coronary inflammation was quantified using the perivascular fat attenuation index (FAI) in the right coronary artery, with high-FAI defined as above the 75th percentile. Multivariable Poisson regression with robust error variance was employed to evaluate the relationship between cancer history and high-FAI, adjusting for conventional cardiovascular risk factors.

**Results:**

The median age and FAI in the overall study population were 70 years and −75.8 HU, respectively. Multivariable analysis revealed a significantly increased prevalence of high-FAI in cancer patients (relative risk [RR]: 1.56; 95% CI: 1.23-1.97) compared to noncancer patients. Stratified analysis based on times after cancer treatment revealed that patients with <5 years postcancer treatment showed a significant association with the prevalence of high-FAI (RR: 1.70; 95% CI: 1.31-2.21) compared to noncancer patients, whereas no significant association was observed between patients with ≥5 years postcancer treatment and high-FAI (RR: 1.29; 95% CI: 0.87-1.92).

**Conclusions:**

In this population, a cancer history, especially in patients with current or recent treatment history, was significantly associated with elevated coronary inflammation.

The global burden of cancer continues to rise, driven by advancements in early detection and therapeutic interventions.^1^^,^^2^ Previous studies have consistently demonstrated that a history of cancer is associated with an increased risk of atherosclerotic cardiovascular events, both during oncologic treatment and in the survivorship phase, across both population-based cohorts and patients with established coronary artery disease.^3^, ^4^, ^5^ As cancer-related mortality declines in parallel with therapeutic progress, cardiovascular mortality has emerged as a leading cause of death among cancer patients,^6^ thereby highlighting the urgent need for comprehensive cardiovascular prevention strategies in this population to optimize long-term outcomes.

Multiple pathophysiological mechanisms have been suggested to explain the increased incidence of atherosclerotic cardiovascular disease in cancer patients.^7^ These encompass shared conventional risk factors, including tobacco use, obesity, hypertension, and diabetes mellitus, which predispose individuals to both malignancy and cardiovascular disease.^8^ The cardiotoxic nature of specific cancer treatments, including chemotherapeutic agents, radiation therapy, and targeted molecular treatments, further exacerbates atherosclerotic progression.^9^ Moreover, persistent systemic inflammation driven by the malignancy itself, particularly through proinflammatory cytokines like interleukin-6 and tumor necrosis factor-alpha, is speculated to play pivotal roles in the pathogenesis of both cancer and atherosclerosis.^9^^,^^10^ However, it remains uncertain whether cancer itself independently contributes to atherogenesis in addition to the established influences of traditional risk factors and treatment-related cardiotoxicity. To date, no previous clinical studies have confirmed a direct association between cancer history and coronary atherosclerotic plaque development.

The fat attenuation index (FAI), a novel imaging biomarker derived from coronary computed tomography (CT) angiography (CCTA), has recently gained attention as a noninvasive indicator of coronary inflammation.^11^ Inflammatory stimuli emanating from the vascular wall induce phenotypic changes in the surrounding pericoronary adipose tissue (PCAT), resulting in a shift in CT attenuation values from lipid-dominant to water-enriched profiles.^12^ By capturing these inflammation-driven modifications in PCAT, FAI enables the quantitative assessment of coronary inflammation, offering a clinically accessible tool for cardiovascular risk stratification using standard CCTA imaging.^11^ Elevated perivascular FAI has been independently associated with accelerated plaque progression and an increased incidence of adverse cardiovascular events, even in patients without obstructive coronary artery disease.^13^, ^14^, ^15^, ^16^, ^17^

Therefore, this study aimed to investigate whether a history of cancer is independently associated with coronary inflammation, as measured by perivascular FAI. Clarifying this relationship is crucial for guiding tailored cardiovascular prevention strategies in the growing population of cancer patients and survivors.

## Methods

### Study populations

**Figure 1:**
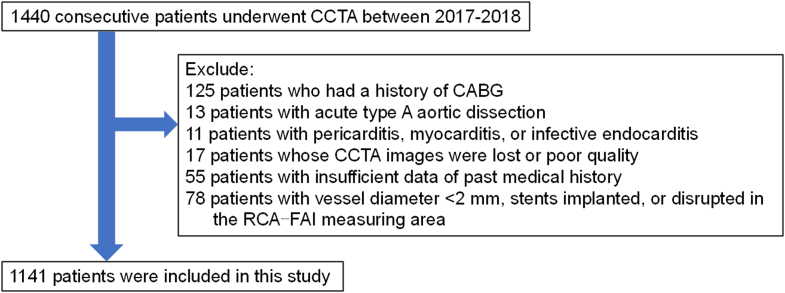
**Flowchart of Subject Selection** CABG = coronary artery bypass grafting; CCTA = coronary computed tomography angiography; FAI = fat attenuation index; RCA = right coronary artery.

This is a retrospective observational study at Aichi Medical University. This study enrolled 1,440 consecutive patients who underwent CCTA for clinical indications at our hospital between 2017 and 2018 (Figure 1). We excluded 125 patients who had a history of coronary artery bypass grafting, 13 patients with acute type A aortic dissection, 11 patients with pericarditis, myocarditis, or infective endocarditis, 17 patients whose CCTA images were lost or poor quality, 55 patients with insufficient data of past medical history, 78 patients with vessel diameter <2 mm, stents implanted, or disrupted in the FAI measuring area. Thus, finally, 1141 patients were included in the analysis. Participants were divided into 2 groups based on cancer history: 953 patients without a history of cancer (noncancer group) and 188 patients with a history of cancer (cancer group).

This study was performed following the principles of the Declaration of Helsinki and was approved by the Institutional Review Board of Aichi Medical University (2024-151). This observational study does not include any invasive procedures or interventions for the participants. Thus, we obtained informed consent for participation verbally whenever possible, or used an opt-out method for participants who could not be contacted directly.

### Definition of variables

Baseline data were collected around the time of CCTA performance. Body mass index was calculated as weight (in kg) divided by height (in m) squared. Hypertension was defined as blood pressure ≥140/90 mm Hg and/or a history of antihypertensive medication use. Diabetes mellitus was defined as a fasting blood glucose level ≥126 mg/dL, glycated hemoglobin (National Glycohemoglobin Standardization Program) ≥6.5%, or a history of antidiabetic medication use. Dyslipidemia was defined as high-density lipoprotein cholesterol <40 mg/dL, low-density lipoprotein cholesterol ≥140 mg/dL, triglycerides ≥150 mg/dL, or a history of lipid-lowering medication use. Current smokers were defined as participants who had smoked within the past 30 days. Patients with any history of cancer were included in the cancer group. Cancer status was determined by reviewing medical records of each patient, with data obtained on cancer treatment details, cancer location, the presence or absence of metastasis, and the time since the completion of cancer treatment. Patients undergoing cancer treatment were defined as those planned for surgery for cancer treatment, receiving chemotherapy and/or radiation therapy, having metastasis, and/or presenting with inoperable cancer. The time since the completion of cancer treatment was defined as the duration until such a condition was overcome. Patients diagnosed with cancer within 6 months following CCTA were also classified in the cancer group, along with patients who were undergoing cancer treatment.

### CCTA image acquisition and analysis

Electrocardiogram-gated CCTA was conducted using a 2 × 64-slice dual-source CT scanner (SOMATOM Definition Flash, Siemens Healthineers). Before the scan, all patients received sublingual nitroglycerin (0.3 mg) to induce coronary vasodilation. For patients with a heart rate exceeding 70 beats/min, intravenous landiolol hydrochloride (Corebeta, Ono Pharmaceutical Co, Ltd) was administered at a dose of 0.125 mg/kg to reduce the heart rate to ≤70 beats/min. A test bolus technique was employed to measure contrast timing. A small amount of contrast agent (iodine dose: 24.5 mg I/kg/s) was injected over 2 seconds, followed by a 25-mL saline flush at the same rate. Low-dose monitoring images were acquired every second at the level of the ascending aorta. The delay time for the main scan was calculated based on the time to peak enhancement, plus an additional 3 seconds. The contrast dose for the main scan was 24.5 mg I/kg/s, injected over 12 seconds, followed by a 25-mL saline flush at the same rate. Iopamidol (Iopamiron 370, Bayer Yakuhin, Ltd.) or iohexol (Omnipaque 350, GE Healthcare Pharma) was administered for contrast-enhanced imaging.

The scan and reconstruction parameters for the main scan were as follows: tube voltage, 120 kV; beam width, 38 mm; detector collimation, 64 × 0.6 mm; pitch factor, 0.17; gantry rotation time, 0.28 seconds; tube current was automatically adjusted between 90 and 1300 mA according to the patient’s body habitus; slice thickness, 0.75 mm; scan field of view, 320 mm; display field of view, 180 mm; matrix, 512 × 512. Image reconstruction using a vascular kernel (Bv41) and a sinogram-affirmed iterative reconstruction (SAFIRE) algorithm with strength level 3.

### FAI measurement

Pericoronary FAI was evaluated using a specialized workstation (Aquarius iNtuition Edition, version 4.4.13.P3A, TeraRecon Inc.). We automatically traced the proximal 40-mm segments of the 3 major epicardial coronary arteries, making manual adjustments when necessary. Following previously validated methods,^16^^,^^18^^,^^19^ we focused on the proximal segments of the right coronary artery (RCA) (10-50 mm from the ostium), the left anterior descending artery (LAD) (0-40 mm from the left main stem bifurcation), and the left circumflex artery (LCX) (0-40 mm from the left main stem bifurcation). PCAT refers to the fat located within a radial distance from the outer vessel wall of 3 mm. Its classification relies on voxels exhibiting attenuation values between −190 and −30 HU.^16^^,^^18^^,^^19^ FAI was determined by averaging the CT attenuation of PCAT, which was reported in HU. All FAI measurements were conducted by a trained investigator blinded to clinical data. Vessels <2 mm in diameter, containing implanted stents, or disrupted within the measurement region were excluded from analysis. Obstructive coronary artery disease was defined as ≥50% stenosis in the left main coronary artery or ≥70% stenosis in any of the 3 major epicardial coronary arteries with a diameter of ≥2 mm.

### Statistical analysis

Continuous variables are represented as the median (IQR), whereas categorical variables are reported as numbers (percentages). We compared continuous variables using the Mann-Whitney *U* test or the Kruskal-Wallis test, and categorical variables were analyzed using Fisher exact test or the chi-square test. In alignment with prior multicenter studies, this analysis used the FAI in the RCA as a primary indicator of pericoronary inflammation,^11^^,^^14^^,^^18^ whereas correlations in other arteries were evaluated as exploratory analyses. Univariable and multivariable linear regression analysis, adjusting for age, sex, body mass index (BMI), current smoking status (yes/no), diabetes mellitus, hypertension, dyslipidemia, and estimated glomerular filtration rate, were performed to examine the association between cancer history and FAI as a continuous outcome. Model assumptions of linearity, independence, homoscedasticity, and normality were evaluated through residual diagnostics and were found to be reasonably satisfied. Subsequently, we defined high FAI as values above the 75th percentile and examined its association with cancer history. Univariable and multivariable Poisson regression with robust error variance was conducted to estimate the relative risks (RRs) and 95% CIs of cancer history regarding high-FAI prevalence. We employed Poisson regression with robust error variance because the prevalence of each indicator exceeded 10% in this study, and interpreting ORs as RRs is inappropriate.^20^ Model 1 was adjusted for age and sex. Model 2 included adjustments for the variables in model 1, BMI, current smoking status (yes/no), diabetes mellitus, hypertension, dyslipidemia, and estimated glomerular filtration rate. A further stratified analysis was performed, where patients were categorized into 3 groups based on the time after cancer treatment completion: noncancer, <5 years post-treatment, and ≥5 years post-treatment. These groups were entered as categorical variables in a single Poisson regression model, with the noncancer group used as the reference category. Spearman correlation analysis was used to analyze the correlation between perivascular FAI values of the RCA, LAD, and LCX. All statistical analyses were 2-sided, and a *P* value of <0.05 was considered statistically significant. CIs were not adjusted for multiple comparisons. Calculations were performed using SPSS Statistics (version 30.0, IBM Corp).

## Results

### Baseline characteristics

Table 1 presents the baseline characteristics of all patients. The median (IQR) age was 70 (60-76) years, with 663 patients (58.1%) being male. RCA-FAI distribution among all participants was slightly positively skewed (Supplemental Figure 1). The median (IQR) RCA-FAI in the overall study population was −75.8 (−80.9 to −70.0) HU. Patients in the cancer group were significantly older and had a lower BMI, along with reduced renal function compared to those in the noncancer group. The median (IQR) time after cancer treatment was 2 (0-8) years, with 66.5% of patients undergoing surgery, 14.9% receiving chemotherapy, and 14.9% receiving radiation therapies. The prevalence of cancer types in the cancer group is shown in Table 2. Prostate cancer was the most prevalent (20.7%), followed by gastric (19.1%) and colorectal cancers (16.5%).

**Table 1:** Baseline Characteristics

	Overall (N = 1,141)	Noncancer (n = 953)	Cancer (n = 188)	*P* Value
Age, y	70 (60-76)	69 (59-75)	74 (69-79)	<0.001
Male, n (%)	663 (58.1)	543 (57.0)	120 (63.8)	0.082
Body mass index, kg/m^2^	23.4 (21.2-25.8)	23.4 (21.3-26.0)	23.0 (20.2-25.1)	0.024
Current smoker, n (%)	177 (15.5)	150 (15.7)	27 (14.4)	0.633
Diabetes mellitus, n (%)	338 (29.6)	291 (30.5)	47 (25.0)	0.129
Glycated hemoglobin, %	5.9 (5.6-6.4)	5.9 (5.6-6.4)	5.9 (5.6-6.4)	0.765
Hypertension, n (%)	898 (78.7)	742 (77.9)	156 (83.0)	0.117
Systolic blood pressure, mm Hg	134 (122-148)	134 (121-149)	136 (124-148)	0.666
Diastolic blood pressure, mm Hg	72 (63-82)	73 (63-82)	69 (61-79)	0.016
Dyslipidemia, n (%)	788 (69.1)	663 (69.6)	125 (66.5)	0.404
Total cholesterol, mg/dL	190 (167-216)	191 (168-217)	188 (165-214)	0.200
LDL-cholesterol, mg/dL	106 (87-126)	107 (88-126)	105 (81-125)	0.117
HDL-cholesterol, mg/dL	53 (44-64)	53 (44-64)	53 (42-65)	0.515
Triglyceride, mg/dL	123 (87-180)	124 (89-180)	108 (77-179)	0.083
Statins, n (%)	426 (37.3)	357 (37.5)	69 (36.7)	0.844
eGFR, ml/min/1.73 m^2^	71.0 (59.0-83.0)	72.0 (60.0-84.0)	65.0 (56.0-78.0)	<0.001
Previous PCI, n (%)	108 (9.5)	87 (9.1)	21 (11.2)	0.382
Obstructive CAD, n (%)	257 (22.5)	209 (21.9)	48 (25.5)	0.280
Years after cancer treatment			2 (0-8)	
Cancer treatment				
Surgery, n (%)			125 (66.5)	
Chemotherapies, n (%)			28 (14.9)	
Radiation therapies, n (%)			28 (14.9)	
Other cancer therapies, n (%)			26 (13.8)	
Metastasis, n (%)			10 (5.3)	

Values are median (IQR) or n (%).

CAD = coronary artery disease; eGFR = estimated glomerular filtration rate; HDL = high-density lipoprotein; LDL = low-density lipoprotein; PCI = percutaneous coronary intervention.

**Table 2:** Prevalence of Cancer Types

Prostate cancer	39 (20.7)
Gastric cancer	36 (19.1)
Colorectal cancer	31 (16.5)
Breast cancer	25 (13.3)
Blood cancer	16 (8.5)
Bladder cancer	15 (8.0)
Lung cancer	15 (7.0)
Ovarian/uterine cancer	9 (4.8)
Oral/laryngeal/pharyngeal cancer	7 (3.7)
Renal cancer	5 (2.7)
Liver/gallbladder cancer	4 (2.1)
Brain cancer	3 (1.6)
Thyroid cancer	2 (1.1)
Esophageal cancer	2 (1.1)
Duodenal cancer	2 (1.1)
Pancreatic cancer	1 (0.5)
Multiple cancers	23 (12.2)

Values are n (%).

### The relationship between cancer history and the prevalence of high-FAI

**Figure 2:**
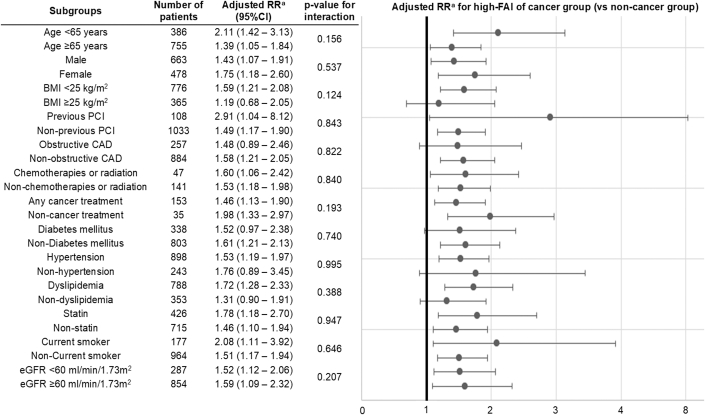
**Adjusted Relative Risk of Cancer History for High-Fat Attenuation Index in Various Subgroups** Consistent association across all subgroups, irrespective of cardiotoxic cancer treatment history or coronary artery disease, was observed. High-FAI was defined as RCA-FAI >75th percentile. ^a^RR was adjusted for age, sex, body mass index, current smoker (yes/no), diabetes mellitus, hypertension, dyslipidemia, and estimated glomerular filtration rate. BMI = body mass index; CAD = coronary artery disease; eGFR = estimated glomerular filtration rate; PCI = percutaneous coronary intervention; RR = relative risk; other abbreviations as in Figure 1.

In univariable analysis, cancer history was associated with higher RCA-FAI (mean difference +1.68 HU; 95% CI: 0.36-3.00; *P* = 0.013). This association remained significant after multivariable adjustment (adjusted mean difference +2.01 HU; 95% CI: 0.70-3.33; *P* = 0.003). Subsequently, we defined high-FAI as values above the 75th percentile, which is >−70.0 HU, and examined its association with cancer history (Table 3). The cancer group showed a significantly higher prevalence of high-FAI compared to the noncancer group (34.0% vs 23.4%, *P* = 0.002). Poisson regression with robust error variance revealed a significant association between cancer history and the prevalence of high-FAI compared to noncancer patients in both univariable (RR: 1.46; 95% CI: 1.16-1.83) and multivariable analysis, adjusting for age, sex, BMI, and other traditional atherosclerotic risk factors (RR: 1.56; 95% CI: 1.23-1.97). Subgroup analysis demonstrated a consistent association between cancer history and high-FAI across all subgroups, regardless of sex, BMI being ≥25 kg/m^2^ or <25 kg/m^2^, prior exposure to cardiotoxic cancer treatments, or the presence of coronary artery disease (Figure 2).

**Table 3:** Associations Between Cancer History and the Prevalence of High-FAI

	Noncancer (n = 953)	Cancer (n = 188)	*P* Value
FAI, HU	−76.1 (−80.9 to −70.5)	−74.0 (−80.9 to −68.3)	0.025
High-FAI, n (%)	223 (23.4)	64 (34.0)	0.002
Univariable RR (95% CI)	Ref	1.46 (1.16-1.83)	0.001
Multivariable (model 1) RR (95% CI)	Ref	1.58 (1.24-2.00)	<0.001
Multivariable (model 2) RR (95% CI)	Ref	1.56 (1.23-1.97)	<0.001

High-FAI was defined as RCA-FAI >75th percentile (>−70.0 HU). Continuous values are expressed as median (IQR).

Model 1 was adjusted for age and sex. Model 2 was adjusted for the variables included in model 1: body mass index, current smoker (yes/no), diabetes mellitus, hypertension, dyslipidemia, and estimated glomerular filtration rate.

FAI = fat attenuation index; HU = Hounsfield unit; RCA = right coronary artery; RR = relative risk.

The perivascular FAI values of the RCA, LAD, and LCX were correlated with each other in Spearman’s correlation analysis (Supplemental Table 1). A significant association was observed between cancer history and the prevalence of high-FAI in the LCX when compared to noncancer patients, similar to the FAI in the RCA. In contrast, no significant association was found between high-FAI in the LAD and cancer patients (Supplemental Figure 2, Supplemental Tables 2 and 3).

### Association between time since completion of cancer treatment and the prevalence of high-FAI

Further stratified analyses were performed based on the times after cancer treatment completion. Years after cancer treatment in patients with <5 years post-treatment ranged from 0 to 4 years, with a median of 0 (IQR: 0-2). For those with ≥5 years post-treatment, the range was from 5 to 40 years, with a median of 11 years (IQR: 8-17) (Supplemental Table 4). The proportion of cancer patients treated with surgery was significantly higher in those with ≥5 years post-treatment compared to those with <5 years (89.9% vs 52.9%, *P* < 0.001). In addition, the proportion of patients who received radiation therapy (7.2% vs 19.3%, *P* = 0.025) and those with metastasis (0.0% vs 8.4%, *P* = 0.013) was significantly higher in patients with <5 years postcancer treatment than in those with ≥5 years post-treatment.

Patients with <5 years postcancer treatment had the highest prevalence of high-FAI (37.8%), followed by those with ≥5 years postcancer treatment (27.5%), and those with noncancer (23.4%, *P* = 0.003). After adjusting for conventional atherosclerotic risk factors, multivariable Poisson regression with robust error variance showed that <5 years postcancer treatment groups had a significantly higher prevalence of high FAI compared to the noncancer group (RR: 1.70; 95% CI: 1.31-2.21). However, no significant association was observed between patients with ≥5 years postcancer treatment and the prevalence of high FAI compared to the noncancer group (RR: 1.29; 95% CI: 0.87-1.92) (Table 4).

**Table 4:** Associations Between Cancer Treatment Status and the Prevalence of High-FAI

	Noncancer (n = 953)	≥5 y Postcancer Treatment (n = 69)	<5 y Postcancer Treatment (n = 119)	*P* Value
FAI, HU	−76.1 (−80.9 to −70.5)	−75.3 (−81.3 to −69.2)	−73.0 (−80.9 to −68.0)	0.049
High-FAI	223 (23.4)	19 (27.5)	45 (37.8)	0.003
Univariable RR (95% CI)	Ref	1.18 (0.79-1.76)	1.62^a^ (1.25-2.09)	<0.001^b^
Multivariable (model 1) RR (95% CI)	Ref	1.30 (0.87-1.93)	1.74^a^ (1.33-2.27)	<0.001^b^
Multivariable (model 2) RR (95% CI)	Ref	1.29 (0.87-1.92)	1.70^a^ (1.31-2.21)	<0.001^b^

High-FAI was defined as RCA-FAI >75th percentile (>−70.0 HU). Continuous values are expressed as median (IQR).

Model 1 was adjusted for age and sex. Model 2 was adjusted for the variables included in model 1, as well as body mass index, current smoking status (yes/no), diabetes mellitus, hypertension, dyslipidemia, and estimated glomerular filtration rate.

Abbreviations as in Table 3.

a *P* < 0.05.

b *P* for trend.

## Discussion

In this study, we demonstrated that individuals with a history of cancer exhibited a significantly higher prevalence of elevated FAI, particularly among those who had completed cancer treatment within the past 5 years. In contrast, no significant association was identified in individuals whose treatment had concluded more than 5 years prior. To our knowledge, this is the first study to evaluate the association between cancer history, stratified by time since treatment completion, and perivascular FAI, a validated imaging biomarker of coronary inflammation and atherosclerotic risk. These results suggest that individuals with a recent history of cancer treatment may be at heightened risk for atherosclerotic disease, underscoring the necessity for rigorous cardiovascular screening and intervention in this population (Central Illustration).

**Central Illustration:**
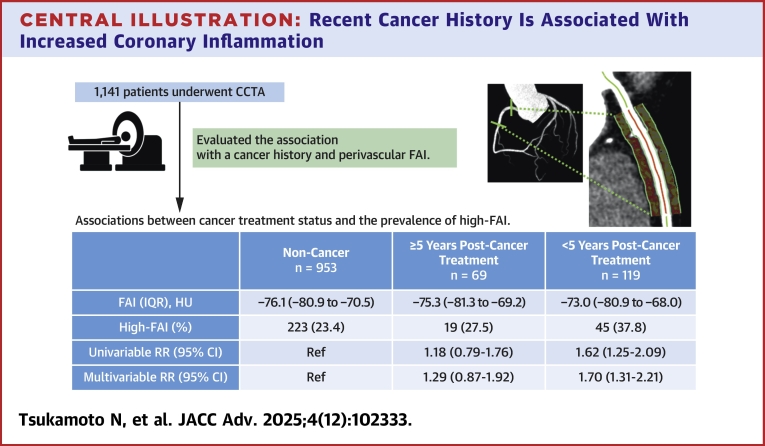
**Recent Cancer History Is Associated With Increased Coronary Inflammation** Our findings indicate that a cancer history, particularly within 5 years following treatment completion, is associated with elevated coronary inflammation as measured by the fat attenuation index (FAI). These findings suggest that individuals with a recent history of cancer treatment might be at an elevated risk for atherosclerotic disease, underscoring the need for close cardiovascular monitoring in this group. High-FAI was defined as FAI in the right coronary artery >75th percentile (>−70.0 HU). CCTA = coronary computed tomography angiography; FAI = fat attenuation index; RR = relative risk.

### Prior evidence linking cancer and cardiovascular risk

A prior study focusing on patients undergoing percutaneous coronary intervention reported that arterial stenosis, as assessed by the ankle-brachial index, and arterial stiffness, as measured by brachial-ankle pulse wave velocity, were significantly associated with a cancer history.^21^ In additionthe , previous studies have consistently identified a history of cancer as an independent risk factor for cardiovascular events across diverse populations.^3^, ^4^, ^5^, ^6^ Notably, the excess cardiovascular risk associated with cancer was reported to be the greatest during the first year following the cancer diagnosis and declined over time. However, it remained significantly elevated for cardiovascular mortality and heart failure even after 10 years of follow-up. Similarly, patients with more advanced cancer were at a higher risk for cardiovascular outcomes.^3^ Given the established association between FAI, coronary plaque progression, and future cardiovascular outcomes,^11^^,^^16^, ^17^, ^18^ and the significantly higher prevalence of metastasis in patients with <5 years postcancer treatment than in patients with ≥5 years postcancer treatment in this study, these prior findings appear to support our results.

## Clinical implementation

As cancer-related mortality continues to decline along with therapeutic advancements, the incidence of cardiovascular events has been increasing,^6^ underscoring the critical importance of integrating cardiovascular care into oncology to optimize overall patient outcomes. Consequently, developing preventive strategies against cardiovascular disease in this population is imperative. Our findings indicate that individuals with a current or recent history of cancer treatment may bear a substantially elevated risk of developing atherosclerotic disease, suggesting the need for comprehensive cardiovascular screening and intervention in this population.

### Association between cancer history and coronary inflammation: future directions

Our study identified a significant association between a history of cancer and elevated FAI, independent of age, sex, BMI, and other established atherosclerotic risk factors. Moreover, this association remained consistent regardless of prior exposure to cardiotoxic therapies such as chemotherapy or radiotherapy, and regardless of the presence of coronary artery disease. These findings might suggest that cancer itself might contribute to the development of atherosclerosis through distinct mechanisms involving chronic inflammation.^7^

This association is particularly pronounced among patients undergoing cancer treatment or within the early post-treatment phase. In contrast, no significant correlation was observed in individuals whose treatment had concluded more than 5 years earlier. The absence of a significant relationship between elevated FAI and patients treated beyond 5 years might reflect a temporal decline in systemic inflammatory activity or successful attenuation of cancer-related inflammatory stimuli. Alternatively, it might suggest a stronger connection between coronary inflammation and active oncologic processes, implying that cancer-associated coronary inflammation might be a reversible and potentially modifiable inflammatory state. Moreover, the emerging evidence indicates that FAI is a dynamic, reversible parameter amenable to therapeutic modulation, such as through statin therapy.^22^ Thus, our findings further raise the possibility that FAI might serve as a valuable noninvasive therapeutic target for quantifying coronary inflammation and assessing cardiovascular risk in patients with cancer.

However, the cross-sectional design of this study constrains the evaluation of temporal changes in FAI in response to effective cancer therapies. Therefore, prospective longitudinal studies are warranted to elucidate the dynamic change of FAI throughout the course of cancer therapy and to validate all the aforementioned hypotheses. Furthermore, interventional studies targeting coronary inflammation, employing FAI as a surrogate endpoint, are necessary to determine whether such an approach can improve cardiovascular outcomes in this population. Nevertheless, the implementation of repeated coronary CT in research settings without a clear clinical indication raises ethical concerns due to the inherent risks of radiation exposure and contrast agent administration. This study could offer a crucial rationale for expanding future research.

Although elevated FAI was more prevalent in cancer patients with BMI <25 kg/m^2^, a similar trend was observed in those with higher BMI, with no significant interaction (*P* = 0.124). Low BMI may reflect greater systemic inflammation due to advanced cancer or cachexia, rather than indicating that obesity is protective. However, only 85 patients (7.4%) had a BMI ≥30 kg/m^2^, and just 15 patients (1.3%) had a BMI ≥35 kg/m^2^ in our study. The small number of obese patients limited our ability to fully evaluate the relationship between obesity and elevated FAI in cancer patients. Further research with a broader range of BMI levels is needed to understand better the complex relationship between adiposity, inflammation, and atherosclerotic risk.

### Artery-specific associations between FAI and cancer history

In the present study, elevated perivascular FAI measurements surrounding the RCA and LCX demonstrated a significant association with a history of cancer. Conversely, no significant association was identified for FAI measured around the LAD. The underlying cause of this discrepancy remains undetermined. Nevertheless, these findings align with those reported in prior studies.^14^^,^^18^^,^^23^ Previous investigations have hypothesized that, unlike the LAD and LCX, the RCA contains a greater volume of perivascular adipose tissue and relatively fewer obstructive nonadipose structures, such as side branches and myocardium, particularly within the proximal segment.^12^^,^^14^^,^^18^ In addition, high-FAI was defined as FAI in the RCA above the 75th percentile (>−70.0 HU) in our population, which also nearly corresponds to the previously reported cutoff value of RCA-FAI ≥ −70.1 HU for cardiovascular mortality.^11^ We also similarly defined high-FAI in the LAD and LCX as those above the 75th percentile for each, since there are no established cutoff values for cardiovascular outcomes in LAD and LCX-FAI. This lack of established thresholds could influence the results of this study.

### Study limitations

Several limitations of this study should be mentioned. First, the results cannot definitively establish causal relationships due to their observational and cross-sectional nature and relatively limited sample size. Second, the possibility of selection bias cannot be dismissed, as the study population consisted solely of patients who underwent CCTA for clinical reasons. Third, the cohort comprised exclusively Japanese patients; given potential variations in cancer prevalence across countries and ethnicities, caution is warranted when generalizing these findings to other populations. Fourth, inflammatory biomarkers such as tumor necrosis factor-alpha and interleukin-6 were not assessed. Fifth, detailed stratified analyses by cancer type or chemotherapeutic regimen were not feasible owing to the limited number of cancer patients included. Finally, CIs were not adjusted for multiple comparisons, which could raise the risk of type I error; therefore, the results should be interpreted cautiously.

## Conclusions

In this cohort, a history of cancer, particularly among individuals undergoing current or recent treatment, was significantly associated with elevated FAI, reflecting increased coronary inflammation.

Perspectives **COMPETENCY IN MEDICAL KNOWLEDGE:** Our study showed that a history of cancer, especially within 5 years after treatment completion, is independently associated with heightened coronary inflammation, as measured by the FAI. Since there is an established correlation between FAI, coronary plaque progression, and adverse cardiovascular outcomes, these results suggest that individuals with recent cancer therapy may have a higher risk of atherosclerosis. This highlights the importance of vigilant cardiovascular screening and intervention.

**TRANSLATIONAL OUTLOOK:** Longitudinal prospective studies are necessary to track changes in FAI throughout cancer treatment. In addition, intervention studies aimed at reducing coronary inflammation, using FAI as a surrogate marker, are vital to determine if such strategies can improve cardiovascular outcomes in this high-risk population.

## Funding support and author disclosures

This study has been supported by Japan Society for the Promotion of Science (10.13039/501100001691JSPS) KAKENHI Grant Number 24K20226 from the Ministry of Education, Culture, Sports, Science and Technology, Japan. The authors initiated and analyzed the present study. The funding source has no role in the study design or collection. The authors have reported that they have no relationships relevant to the contents of this paper to disclose.
