# Supplementary material for: Association Between Cancer Treatment History and Coronary Inflammation
Source: JACC Adv. 2025 Nov 19;4(12):102333. doi: 10.1016/j.jacadv.2025.102333 (PMC12793851; doi:10.1016/j.jacadv.2025.102333)
Supplement: Supplemental Tables 1 to 4 and Supplemental Figures 1 and 2 [file mmc1.pdf]

Figure legends.

Supplemental Figure 1. Distribution of RCA-FAI.

FAI, fat attenuation index; HU, Hounsfield unit; RCA, right coronary artery.

Supplemental Figure 2. Flowchart of subject and vessel selection.

CABG, coronary artery bypass grafting; CCTA, coronary computed tomography angiography; FAI, fat attenuation index; LAD, left anterior descending artery; LCX, left circumflex artery; RCA, right coronary artery.

Supplemental Figure 1. Distribution of RCA-FAI.

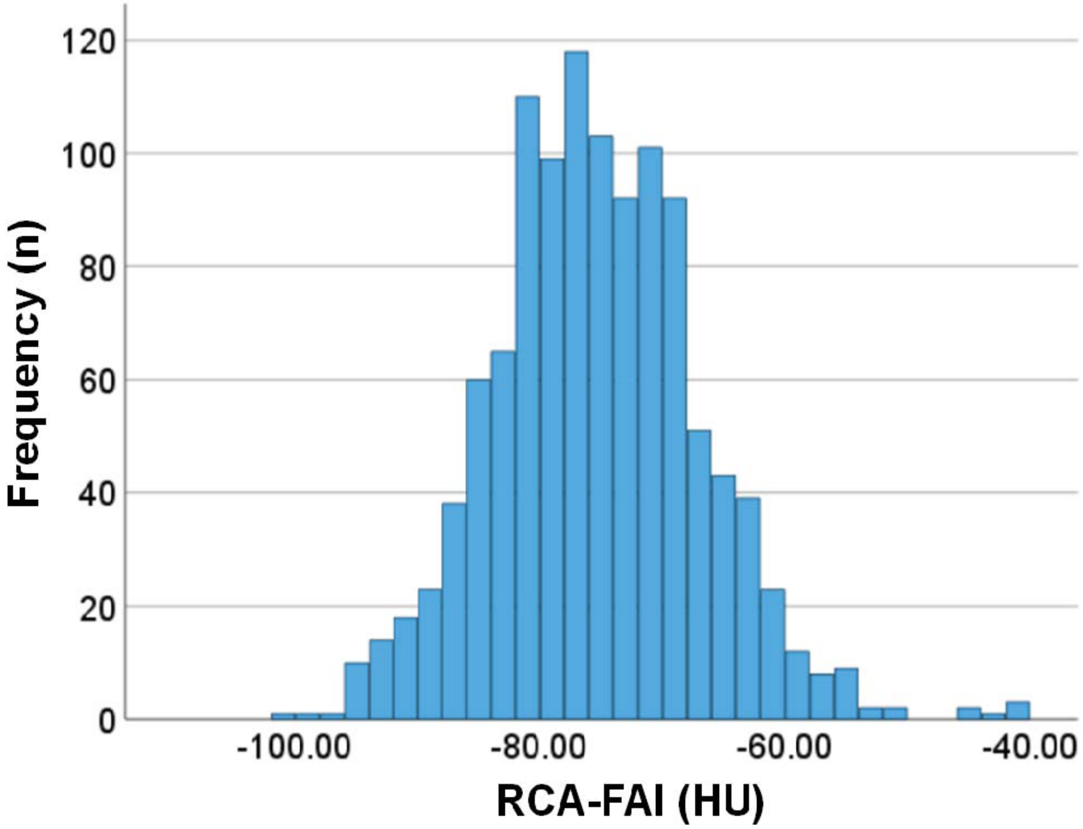

Supplemental Figure 2. Flowchart of subject and vessel selection.

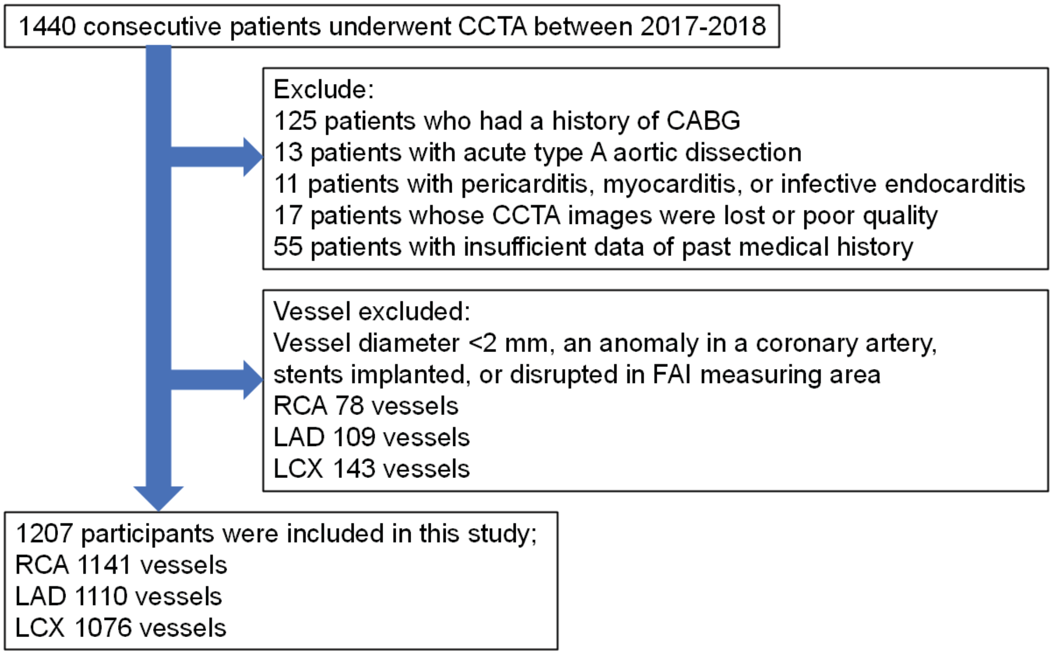

Supplemental Table 1. Correlations of FAI among the three coronary arteries.

Variables	ρ	*p*-value
RCA-FAI vs LAD-FAI	0.51	<0.001
RCA-FAI vs LCX-FAI	0.58	<0.001
LCX-FAI vs LAD-FAI	0.63	<0.001

Correlations were analyzed using Spearman's correlation analysis. FAI, fat attenuation index; LAD, left anterior descending artery; LCX, left circumflex artery; RCA, right coronary artery.

Supplemental Table 2. The number (percentages) of high LAD and LCX-FAI in each cancer group

	Non-cancer<br>n = 931	Cancer<br>n = 179	*p*-value
LAD-FAI, HU	-73.1 (-78.4 – -67.8)	-72.8 (-78.3 – -67.0)	0.282
High LAD-FAI, n (%)	226 (24.3)	52 (29.1)	0.177
	Non-cancer<br>n = 898	Cancer<br>n = 178	*p*-value
LCX-FAI, HU	-70.9 (-75.6 – -65.2)	-68.4 (-74.9 – -62.8)	0.004
High LCX-FAI, n (%)	210 (23.4)	59 (33.1)	0.006

High LAD and LCX-FAI were defined as values greater than the 75th percentile, respectively (-67.6 HU in the LAD and -64.9 HU in the LCX). Continuous values are expressed as median (interquartile range). Categorical values are expressed as numbers (percentages). FAI, fat attenuation index; HU, Hounsfield Unit; LAD, left anterior descending artery; LCX, left circumflex artery.

Supplemental Table 3. Associations between cancer history and the prevalence of high LAD and LCX-FAI.

	Univariable RR (95% CI)	Multivariable (Model 1) RR (95% CI)	Multivariable (Model 2) RR (95% CI)
LAD			
Non-cancer n = 931	Ref	Ref	Ref
Cancer n = 179	1.20 (0.93 – 1.55)	1.08 (0.83 – 1.40)	1.03 (0.80 – 1.33)
LCX			
Non-cancer n = 898	Ref	Ref	Ref
Cancer n = 178	1.42[a] (1.12 – 1.80)	1.42[a] (1.11 – 1.83)	1.38[a] (1.07 – 1.78)

[a] p <0.05

High LAD and LCX-FAI were defined as values greater than the 75th percentile, respectively (-67.6 HU in the LAD and -64.9 HU in the LCX). Model 1 was adjusted for age and sex. Model 2 was adjusted for the variables included in Model 1, as well as body mass index, current smoking status (yes/no), diabetes mellitus, hypertension, dyslipidemia, and estimated glomerular filtration rate. CI: confidence interval; FAI, fat attenuation index; LAD, left anterior descending artery; LCX, left circumflex artery; RR, relative risk.

Supplemental Table 4. Baseline characteristics of patients stratified by cancer treatment status.

	Non-cancer<br>n = 953	≥5 years post-cancer treatment<br>n = 69	<5 years post-cancer treatment<br>n = 119	*p*-value
Age, years	69 (59 – 75)	74 (70 – 79)	74 (68 – 78)	<0.001
Male, n (%)	543 (57.0)	39 (56.5)	81 (68.1)	0.067
Body mass index, kg/m^2	23.4 (21.3 – 26.0)	23.1 (20.1 – 26.0)	23.0 (20.6 – 24.8)	0.076
Current smoker, n (%)	150 (15.7)	11 (15.9)	16 (13.4)	0.804
Diabetes mellitus, n (%)	291 (30.5)	13 (18.8)	34 (28.6)	0.117
Glycated haemoglobin, %	5.9 (5.6 – 6.4)	6.0 (5.7 – 6.4)	5.9 (5.6 – 6.4)	0.504
Hypertension, n (%)	742 (77.9)	57 (82.6)	99 (83.2)	0.292
Systolic blood pressure, mmHg	134 (121 – 149)	136 (124 – 148)	136 (123 – 148)	0.893
Diastolic blood pressure, mmHg	73 (63 – 82)	69 (60 – 80)	71 (62 – 79)	0.041
Dyslipidemia, n (%)	663 (69.6)	45 (65.2)	80 (67.2)	0.677
Total cholesterol, mg/dL	191 (168 – 217)	188 (166 – 219)	188 (161 – 212)	0.372
LDL-cholesterol, mg/dL	107 (88 - 126)	97 (78 – 125)	106 (77 – 176)	0.221
HDL-cholesterol, mg/dL	53 (44 – 64)	54 (47 – 66)	52 (41 – 64)	0.242
Triglycerides	124 (89 – 180)	108 (80 – 191)	106 (77 – 176)	0.372
Statins, n (%)	357 (37.5)	25 (36.2)	44 (37.0)	0.976
eGFR, ml/min/1.73m^2	72.0 (60.0 – 84.0)	63.0 (56.0 – 80.5)	66.0 (56.0 – 77.0)	0.001
Previous PCI, n (%)	87 (9.1)	8 (11.6)	13 (10.9)	0.675
Obstructive CAD, n (%)	209 (21.9)	14 (20.3)	34 (28.6)	0.237
Years after cancer treatment				
Median (interquartile range), years		11 (8 – 17)	0 (0 – 2)	<0.001
Minimum – Maximum, years		5 – 40	0 – 4	
Cancer treatment				
Surgery, n (%)		62 (89.9)	63 (52.9)	<0.001

Chemotherapies, n (%)		10 (14.5)	18 (15.1)	0.906
Radiation therapies, n (%)		5 (7.2)	23 (19.3)	0.025
Other cancer therapies, n (%)		6 (8.7)	20 (16.8)	0.120
Metastasis, n (%)		0 (0.0)	10 (8.4)	0.013

Continuous values are expressed as median (interquartile range). Categorical values are expressed as numbers (percentages). We compared continuous variables using the Kruskal–Wallis test, and categorical variables were analyzed using Fisher's exact test or the chi-squared test. CAD, coronary artery disease; eGFR, estimated glomerular filtration rate; HDL, high-density lipoprotein; LDL, low-density lipoprotein; PCI, percutaneous coronary intervention.
